# Instruments for evaluating the parental emotional status and ecological support systems among parents who considered cochlear implantation for their children with hearing loss: A scoping review

**DOI:** 10.1371/journal.pone.0305748

**Published:** 2024-07-29

**Authors:** Tang Zhi Lim, Cila Umat, Pei-Hua Chen, Chun Hong Gan, Bee See Goh

**Affiliations:** 1 Center for Rehabilitation & Special Needs Studies, Faculty of Health Sciences, Universiti Kebangsaan Malaysia, Kuala Lumpur, Malaysia; 2 Speech and Hearing Science Research Institute, Children’s Hearing Foundation, Taipei, Taiwan; 3 Department of Otorhinolaryngology and Head and Neck Surgery, Faculty of Medicine, Universiti Kebangsaan Malaysia, Kuala Lumpur, Malaysia; 4 Hospital Canselor Tuanku Muhriz, Kuala Lumpur, Malaysia; Lamar University, UNITED STATES

## Abstract

**Objectives:**

Parents of children diagnosed with severe-to-profound sensorineural hearing loss may experience a range of emotions owing to a lack of knowledge and experience in dealing with such children. However, most audiology clinics only attend to children with deaf and hard of hearing (DHH) and not their parents. Thus, parents’ emotional and support needs are frequently excluded from the intervention sessions, making their own needs invisible. This study aimed to identify academic and clinical instruments used for assessing parental emotional status (PES) and ecological support systems (ESS) in early intervention and determine the factors affecting PES and ESS among parents of DHH children undergoing cochlear implantation.

**Materials and methods:**

This scoping review followed the rigorous methodological framework; searched Medline (via OVID and EMBSCO), Scopus, and Web of Science; and selected studies relevant to validated instruments used to evaluate the PES and ESS among parents of DHH children below 6 years old. Before selecting and reviewing relevant articles, two reviewers independently assessed article titles and abstracts from the data sources. Two reviewers verified half of the first reviewer’s extracted data.

**Results:**

Overall, 3060 articles were retrieved from the database search, and 139 were selected for full-text review following title and abstract reviews. Ultimately, this study included 22 articles. Among them, 23 and 12 validated instruments, most of which are generic measures, were used for assessing PES and ESS, respectively. Three condition-specific instruments were identified and designed to be administered following cochlear implantation surgery.

**Conclusions:**

This study revealed that healthcare professionals who interact with parents of DHH children lack the necessary instruments, particularly for parents of children undergoing cochlear implantation surgery. Therefore, it is necessary to develop condition-specific instruments for parents who consider cochlear implantation for their children.

## Introduction

Based on previous nationwide studies, the prevalence of hearing loss among newborns is significant compared with other disabilities, accounting for 1–3 in every 1,000 newborns [1–5]. According to Mazlan et al. [3], from 2010 to 2019, the prevalence of hearing loss among newborns in Malaysia was comparable to that in the United States [5] and Singapore [2]. Data indicated that the median age of deafness diagnosis for children with deaf and hard of hearing (DHH) was approximately 3.8 months. However, compared to the recommendations of the Joint Committee of Infant Hearing Position Statement, the relatively late age at which children are fitted with hearing aids or undergo cochlear implant (CI) surgery following a hearing loss diagnosis is concerning [6]. Previous studies conducted in Malaysia [7, 8] reported that the average age of children fitted with hearing aids was approximately 27.5 months, whereas those who underwent CI surgery were between 39.8 and 41.5 months. The disparity between the age of deafness diagnosed and the age of hearing devices fitted raises questions about whether the postdiagnosis experiences of children and their families render such a significant difference. Therefore, despite universal neonatal hearing screening, the lack of awareness regarding the impact of hearing loss on children and the benefits of intervention for families and children’s development has led to a low level of parental engagement and intention to enroll in interventions as early as possible. Consequently, while promoting timely access to early intervention services for children, considering how to assist parents internally and externally to reduce the age difference between diagnosis and intervention is necessary.

Approximately 90% of DHH children are born to hearing parents [5, 9]. Parents’ understanding regarding hearing loss and hearing devices is frequently lacking, as most of them were raised in a hearing community. A study by Wong et al. [10] indicated that mothers or expectant mothers in Malaysia have insufficient knowledge regarding childhood hearing loss. As hearing loss is an invisible disability, parents will fear and ultimately refuse to take any interventional or medical action for their children as it will label their children as individuals with a hearing disability and exclude them from the hearing community [11–14]. Consequently, the parents may hold a false belief that their children will eventually regain their ability to hear, which in turn delays the fitting of hearing devices (both hearing aids and CI) for their children [12, 15–18].

Furthermore, these parental practices may be because of a stigmatized perception and insufficient awareness of hearing. When healthcare professionals inform parents of their child’s deafness, the parents may experience shame and guilt [12, 15–17]. They may question and reject the diagnosis results once they observe their child responding to sounds at home, which contradicts the diagnosis results [12, 18, 19]. Therefore, parents would dispute the hearing loss diagnosis with the healthcare professionals and believe their child is too young to be examined [20]. Moreover, parents may believe that having a child with DHH is a curse because they may have performed something wrong during pregnancy, and now their children are suffering the consequences [12, 21–23], especially for non-Malay parents in Malaysia [10]. Therefore, parents who are not emotionally ready may experience parental burnout, which in turn delays their child’s intervention process [24]. However, rather than being overwhelmed by negative emotions, some parents are proactive and persistent in finding a solution for their children upon learning of the diagnosis from the healthcare professional [25]. Instead of getting trapped in an emotional hole, they would comprehend what to do for their children; therefore, their children are enrolled in the intervention as early as possible. Further, some parents may initially show negative emotions; however, when their children communicated with them after undergoing interventional sessions, these parents experienced a positive shift and overcame their distress [25]. Therefore, regardless of parents’ personalities and initial reactions, their awareness of the urgency of taking the next step for their children will affect the timing of their access to the intervention.

Furthermore, apart from the inherent factors, such as the parents’ restricted emotional regulation ability and hearing loss awareness, external (resource) factors, such as ecological support systems (ESS), may also play a role in the delayed access to intervention resources experienced by the family. The provision of ESS, whether received from family, friends, support groups, or healthcare professionals, plays a crucial role in assisting parents as they access the intervention process, regardless of the communication modalities or hearing devices they select [26]. For example, caregivers receiving sufficient family and partner support during intervention sessions will influence the caregivers’ reactions and behaviors through those sessions [27–29]. The CI intervention journey requires not only considerable time and effort to attend various medical appointments but also a potential financial burden. Devoid of assistance from partners and family members, the parent’s emotional state may become overwhelming. Moreover, healthcare professionals play a role in not only offering the necessary professional services but also creating a space for parents to express their feelings [30]. This aspect will effectively support and motivate parents to accompany their children throughout the intervention process [29, 31]. Nevertheless, previous studies revealed that inconsistencies in the guidance offered by professionals have been experienced by parents. These inconsistencies have resulted in delays in their attempts to intervene and have hindered the prompt fitting of hearing devices and communication modality program implementation for their DHH children [12, 19, 32]. This situation may be particularly present in children with additional disabilities [33]. A study by Hamzah et al. [12] reported that some parents noted that their child’s development differed from that of other children; therefore, they brought their child to professionals for further examination. However, the parents were advised not to worry because the child was too young and needed to wait and observe how things progressed without undergoing any examination. This finding is consistent with a study that noted that 75.4% of primary care physicians in public health clinics in Malaysia lacked knowledge of hearing issues and did not consider hearing problems to be a significant health issue [34]. Therefore, instances wherein healthcare professionals lack knowledge of childhood hearing loss contribute to the delay in the initiation of the intervention process. Consequently, the emotional state, attitude, and knowledge of parents regarding hearing loss, along with ESS, may significantly affect the timely intervention of a child with DHH.

Considering the abovementioned perspective, the clinical settings, and evidence derived from previous studies conducted in Malaysia, which indicated a discrepancy in age between diagnosis and hearing device fitting [7, 8], several factors that may have been contributing to these discrepancy circumstances require attention. Although previous studies demonstrated the beneficial impact of early intervention on the future development of DHH children [35, 36], considering the parental emotional status (PES) and the available support (ESS) that parents had during the phase preceding the intervention period is important. Parents may have experienced challenging circumstances and encountered emotional allegations from others, which may have caused them to be hesitant to take further action. For instance, some people might criticize the parents, attributing the child’s deafness to a negative action that was done by the mother during pregnancy and considering it as a manifestation of her karma [12, 17]. Furthermore, following CI surgery, parents express concerns regarding the potential benefits their children may derive from the implants. They may experience negative feelings not only in the pre- and postoperative periods but also in the long-term reliability of CI use and intervention sessions [30].

Several studies have shown that early intervention that focuses on spoken language acquisition, particularly before a child reaches 6 months old, leads to better speech and language development [35, 37]. However, initiating early intervention can be challenging when parents are not mentally prepared, particularly if the child is a suitable candidate for a CI, which can be a challenging decision for them [30]. Therefore, considering not only the child’s hearing level and the suitability of hearing devices but also whether the parents are mentally prepared for their child’s intervention is imperative. In this regard, healthcare professionals require the proper instruments or competencies to motivate parents to expedite parental decision making regarding early intervention [30, 38]. Therefore, this study aimed to identify suitable instruments for evaluating the parents’ PES and their ESS and compile the key variables influencing their emotional state and the support they receive with respect to early diagnosis and intervention for DHH children, specifically for parents of children undergoing cochlear implantation.

## Materials and methods

This scoping review study was conducted and reported according to the Preferred Reporting Items for Systematic Reviews and Meta-Analyses Extension for Scoping Reviews (PRISMA-ScR) guidelines [39–41].

### Eligibility criteria

The population, concepts, and context approach were referred to when developing the inclusion criteria to address the research questions [41, 42]. First, for the type of participants (population), although the studies focused on parents of DHH children, in terms of early intervention, the inclusion criteria would be for children falling within the chronological age range of newborn to preschool. Specifically, parents whose children’s chronological ages ranged from 0 to 6 years would be included. Additionally, parents whose children underwent CI surgery before 6 years old, regardless of their current chronological age, would be included. Studies including children with prelingual or late-onset deafness, hearing aid users, CI users, or CI candidates were included. Second, the concept may comprise details pertaining to instruments that evaluate “parental emotional status,” and/or “parental ecological support systems,” and/or “parental quality of life.” Lastly, the context must be English-language-only peer-reviewed publications with no date restrictions, and the articles discussed the PES and/or parental support systems and/or parental quality of life among parents of DHH children using validated instruments.

### Types of evidence sources

This study included only peer-reviewed articles that were written in English with no date restrictions. Furthermore, pre-prints, pilot studies, case studies, protocol articles, proceeding articles, literature review articles, systematic reviews, or scoping reviews were not included in this study. Qualitative studies that do not use validated instruments were excluded from the present study. Therefore, only studies that used validated instruments and had parents of DHH children, whose chronological age ranged from 0 to 6 years or had undergone CI surgery between 0 and 6 years old were included. More specifically, articles that relate to parents of preschoolers/toddlers/infants who have hearing loss (either congenital or late-onset hearing loss) were included in this study.

### Information sources

To discover articles pertinent to the research question, this study used Medline (via OVID and EMBSCO), Scopus, and Web of Science (WOS). Before entering the article title and abstract screens, articles that discussed adults and/or did not include hearing loss were excluded. The bibliographic databases were searched in March 2023.

### Search

Medical subject headings and keywords for the search were covered in the following three key domains:

1. Population: parents of DHH children.
2. Measurement: questionnaire, scale, survey, inventory, or instrument.
3. Concept: quality of life, emotional state of parents, or ESS.

The full electronic search strategy comprised (parent* OR father* OR mother* OR caregiver* OR famil*) AND (children with hearing loss OR children with hearing impairment OR deaf children OR children with deaf or hard of hearing) AND (scale* OR test* OR questionnaire* OR assessment* OR measure* OR inventor* OR instrument*) AND (parent emotion* OR parental emotional readiness OR parental support OR parental support system* OR parental ecological support system* OR parental ecological support OR quality of life). Although the input of Boolean and search terms varies from database to database, it will be adjusted according to the system’s interface and input method; however, the relevant Boolean and keyword searches are clearly linked to the three key domains.

### Selection of sources of evidence

**Fig 1:**
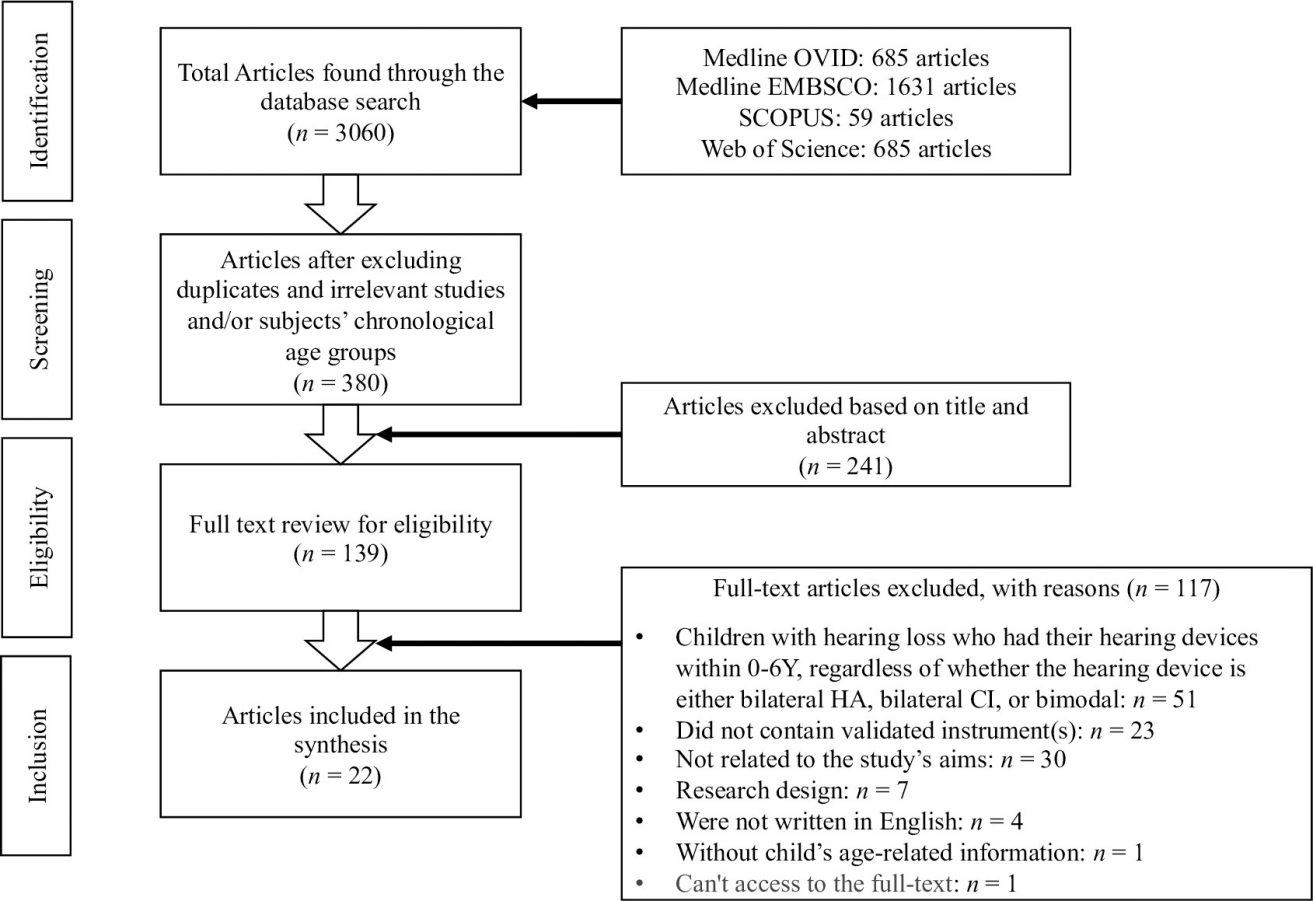
Flowchart of the article review process (PRISMA-ScR).

The first author performed a literature search for potentially relevant publications measuring PES and ESS among children with hearing loss. Before the first and second authors separately assessed the titles and abstracts of the original articles (proportionate agreement = 0.82), the first author screened and excluded non-hearing loss-related articles after deleting duplicate publications. Extracted studies that used validated instruments for evaluating parental outcomes (either PES or ESS) among DHH children who had their hearing devices at the age of 0–6 years regardless of whether the hearing device was either bilateral hearing aids, bilateral CI, or bimodal were included, and subsequent full-text data extraction finalized the application of the inclusion criteria. During the full-text review phase, the fourth author crosschecked approximately 20% of the articles (proportionate agreement = 0.84). Disagreements over eligibility were addressed by obtaining a third opinion from the third author in both the title and abstract screening and full-text review phases (Fig 1). The review process was performed using the Covidence software during the title and abstract screening and full-text reviews, whereas Microsoft Excel was used during the data extraction phase. Covidence provides the ideal user experience for researchers conducting a systematic/scoping review during the title and abstract screening phase [43].

### Data charting

The present database inquiry was conducted between February and March 2023. The first author developed the data-charting table (in Microsoft Excel) for use in the full-text review section and sent it to the second and third authors for additional review and correction while checking the title and abstract. Once the data extraction table’s initial iteration was completed, the second and third authors revised it as necessary.

### Data items

To characterize the data from the included articles, a set of variables was defined prior to their extraction for this study. After the second and third authors reviewed the data-charting table, the extracted information was inserted into the table according to the variables listed in the Microsoft Excel sheet. Author(s) and publication year, research design, participant characteristics number of participants, gender of the parents and children, and age-related variables (including child’s age of deafness diagnosis, chronological age, and age at cochlear implantation), child’s device setting, objective(s) of the study, instrument(s) used, and outcomes were listed as variables. This information would summarize existing instruments for evaluating PES and ESS among parents of DHH children.

### Synthesis of results

The data and findings from the included studies are integrated and presented in Table 1.

**Table 1:** Characteristics of the included studies on parental emotional status and ecological support systems among parents of DHH children.

ID	Author(s) & Publication Year	Research Design	Participant Characteristics				Child’s Device Setting	Objective(s)
			Number of Parents and/or Children	Gender (Parents)	Gender (Children)	Child’s Age-Related Characteristics (Mean Age [SD; minimum–maximum])		
1	Aiello & Ferrari. 2015. [44]	Case–control study	22 mothers of DHH children (11 each for the control and experimental groups)	22 hearing mothers	10 males (5 each for the control and experimental groups) 12 females (6 each for the control and experimental groups)	Age of deafness diagnosis (months): 10.2 (8.3; did not mention); Child’s chronological age (months): 20.3 (6.9; did not mention)	All CI candidates	(1) To assess the parental stress of parents of DHH children who were CI candidates (2) To evaluate the efficacy of the online social network “Babies’ Portal” as an instrument for supporting these parents
2	Aras et al. 2014. [45]^$^	Case–control study	626 parents of children with (1) speech impairment (receptive–expressive language disorder; SI), (2) severe hearing impairment (DHH), and (3) control group	SI: 349 parents (182 mothers and 167 fathers) DHH: 131 parents (71 mothers and 60 fathers) Control group: 146 (82 mothers and 64 fathers)	Did not mention	Preschool-aged children	Most of these children used CI, and the rest relied on hearing aids.	To estimate the expected negative influence of children’s speech and hearing problems on their parents’ subjective health by measuring parental health-related quality of life and focusing on the physical, emotional, and social aspects of this impact
3	Brand et al. 2018. [46]	Cross-sectional study	70 Jewish married couples (father–mother pairs) of children with mild-to-profound hearing loss	70 hearing fathers and mothers	32 males 37 females	Age of deafness diagnosis (months): 13.9 (12.8; did not mention); Child’s chronological age (years): 5.25 (1.6; did not mention)	18 HAs 51 CIs	(1) To examine the relationship between parents’ individual coping resources and their involvement. Specifically, to examine whether mothers and fathers self-reported coping resources will be positively associated with their report on involvement (2) To examine the relationship between mothers’ coping resources and fathers’ involvement and the relationship between fathers’ coping resources and mothers’ involvement (3) To examine whether parental gender and the family’s level of religiosity will moderate these actor–partner effects
4	Cejas et al. 2021. [47]	Cross-sectional study	164 children with bilateral severe-to-profound sensorineural hearing loss who had 3 years of experience with a CI. Child must be educated in English-speaking schools	Did not mention	80 males 84 females	Age of hearing loss onset (months): 2.7 (7.3; 0–44); Age of CI (months): 28.8 (did not mention; 6–64); Child’s CI activation age (years): 2.4 (1.2; 0.6–5.3)	All CI users	To evaluate associations between parenting stress, self-efficacy, and involvement with respect to spoken language outcomes in young children aged 3 years following cochlear implantation
5	Cejas et al. 2022. [48]^#^	Cross-sectional study	110 parents of children with CI. Open-ended interview: 19 parents of children with CI Cognitive testing: 19 parents of children with CI Psychometric validation: 72 parents of children with CI	Open-ended interview: 17 mothers (89.5%) and 2 fathers (10.5%) Cognitive testing: 15 mothers (78.9%) and 4 fathers (21.1%) Psychometric validation: 68 mothers (94.4%) and 4 fathers (5.6%)	Did not mention	Child’s chronological age (years): Open-ended interview: 4.29 (1.29; did not mention) Cognitive testing: 3.46 (1.36; did not mention) Psychometric validation: 3.27 (1.45; did not mention)	Open-ended interview + cognitive testing: all CI users Psychometric validation: 11 unilateral CIs 53 bilateral CIs	To develop and validate a CI-specific parenting stress measure for assessing parental needs and individualize family-centered interventions using the FDA Guidance on Patient-Reported Outcomes (Food and Drug Administration [FDA], 2009) for parents of young (aged 0–5 years) and SA children (aged 6–12 years)
6	Dirks et al. 2016. [49]	Case–control study	30 children with moderate hearing loss (MHL) and 30 hearing children (NH)	MHL: 6 fathers and 2 mothers with moderate hearing loss; 1 father was deaf. NH: hearing parents	MHL: 11 males; 19 females NH: 17 males; 13 females	Child’s chronological age (months): MHL: 27.7 (5.6; 18–33) NH: 26.5 (6.5; 17–33) Intervention-related (MHL only) Child’s age at the start of the family intervention (months): 8.3 (7.5; 1–25); Child’s age at hearing aid fitted (months): 9.4 (9.1; 1–33)	All HA users	(1) To examine the level of perceived parental stress in parents of young children with MHL compared with parents of hearing children (2) To explore the associations between parental stress and child- and parent-related factors including language ability, social–emotional development, and social support in children with MHL and their hearing peers
7	Dirks & Szarkowski, 2022. [50]	Cross-sectional study	24 Dutch couples with at least one child with moderate-to-profound hearing loss	24 Dutch couples: All fathers and mothers have typical hearing, except 3 fathers and 1 mother with hearing loss	14 males 10 females	Child’s chronological age (months): 27.38 (12.92; 9–48)	1 ABI 13 CIs 7 HAs 3 BAHAs	To examine the similarities and differences between fathers and mothers of young DHH children related to parental self-efficacy and involvement
8	Gascon-Ramos et al. 2010. [51]	Cohort study	82 families provided initial data (T0), 52 of whom provided follow-up data 6 months later (T1), and 23 of whom also provided follow-up data at 12 months after study entry (T2)	8 parents with disability and 5 parents with deafness	Did not mention	Child’s chronological age (months): 11.7 (6.3; 0.6–27)	Did not mention	To report the results from one section of the My Views on Services (MVOS) concerning the content of intervention in a study including 82 families of early identified children with deafness to understand how parents’ values, beliefs, and preferences mediate the nature of the intervention
9	Green. 2020. [52]	Cohort study	27 parents of children aged 3–5 months who were diagnosed with bilateral hearing loss	2 hearing fathers (1 as the main caregiver) and 25 hearing mothers	Did not mention	Child’s chronological age (months): 3–5 months	Did not mention	To investigate parental adjustment to the permanent bilateral hearing loss diagnosis in infancy and subsequent timing of engagement in medical investigations and early intervention services
10	Hintermair & Sarimski. 2019. [53]	Cross-sectional study	92 fathers of very young DHH children	88 hearing fathers and 4 fathers with hearing loss	41 males 50 females 1 missing data	Child’s chronological age (months): 26.0 (9; did not mention); Child’s age at early intervention (months): 10.3 (6.7; did not mention)	CI: 39	To promptly discover how fathers of infants and toddlers with DHH cope with their situation and how they evaluate their experiences as fathers of a child with DHH
11	Jackson et al. 2010. [54]	Cross-sectional study	Families of 207 DHH children aged 0–6 years	186 hearing mothers 16 hearing fathers 3 hearing grandparents 2 others (Did not mention in the study)	Did not mention	Child’s chronological age (months): 44.0 (16.58; 2–72)	95 HAs 103 CIs 5 no devices 4 missing data	To investigate families’ perceptions of their quality of life following the early identification of deafness in their child to determine key areas of desired family support and provide recommendations for program enhancement
12	Kobosko et al. 2021. [24]	Cross-sectional study	64 hearing mothers of children with bilateral severe or profound sensorineural hearing loss	64 hearing mothers	29 males 35 females	Age of deafness diagnosis (months): 1.0 (1.9; did not mention); Child’s chronological age (months): 23.6 (10.1; 6.5–47); Child’s age of CI1 (months): 14.81 (5.21; did not mention); Child’s age of CI2 (months): 31.71 (9.84; did not mention)	36 CIs (7 bilateral CIs) 28 HAs	To discover whether the different levels of global psychomotor development in young children with deafness who had a CI or were candidates for a CI were related to particular family factors: the self-perceived parental role and the family quality of life as gauged by their hearing mothers, as well as the child’s sociodemographic and deafness-related factors and the mothers’ sociodemographic characteristics
13	Kushalnagar et al. 2007. [55]	Cross-sectional study	46 children with severe-to-profound hearing loss	All hearing parents	25 males 21 females	Age of deafness diagnosis (months): 12.0 (10.8; did not mention); Child’s chronological age (months): 38.9 (27.0; did not mention); Child’s age at the time of enrollment in the CI program (months): 18 (10.9; 3–35)	All CI users	To determine whether adaptive behavior in precochlear implant candidates was correlated with intelligence and parental depression after controlling for early intervention and neurological status
14	Majorano et al. 2020. [56]	Cross-sectional study	20 mothers of children with CIs	20 hearing mothers	Did not mention	Age of deafness diagnosis (months): 8.20 (9.41; 2–28); Child’s age of CI (months): 17.4 (8.38; 1.10–35)	19 Bimodal HAs 1 Bilateral CI	To explore the relationship between three parameters including mothers’ emotional experiences preoperatively, mothers’ stress levels, and their children’s language and communication development
15	Mavrogianni & Lampropoulou. 2020. [57] ^$^	Cross-sectional study	172 fathers of preschool-aged children; DHH children: 25 (both spoken and sign language) children with intellectual disability: 23 children with autism spectrum disorder: 30 children without disabilities: 94	172 hearing fathers	Did not mention	Child’s chronological age (years): 3.51 (1.01; did not mention)	Did not mention	To explore the following: (a) fathers’ involvement with their preschool DHH children compared with the childcare involvement of fathers of children with other disabilities (autism spectrum disorder and intellectual disability) and those without disabilities; (b) the marital satisfaction of fathers of children with deafness compared with that of fathers of children with other disabilities and those without disabilities; (c) the stress experienced by fathers of children with deafness compared with that experienced by fathers of children with other disabilities and those without disabilities; (d) the beliefs concerning the parental role of fathers of children with deafness compared with those of fathers of children with other disabilities and those without disabilities; and (e) the social support reported by fathers of DHH children compared with that of fathers of children with other disabilities and those without disabilities
16	Munoz et al. 2015. [58]	Cross-sectional study	37 English-speaking families with children aged from birth to 3 years with bilateral hearing loss who were using hearing aids	35 hearing mothers 20 hearing fathers	19 males 14 females	Age of deafness diagnosis (months): 3 (5.69; did not mention); Child’s chronological age (months): 22 (8.07; 0–22); Child’s age at hearing aids fitted (months): 7 (6.40; 1–31)	All HA users	To investigate challenges experienced by mothers and fathers with respect to confidence with skills, perception of benefit, hearing aid management, and the influence of challenges on hearing aid use, as well as parental psychosocial characteristics during the first 3 years of life in children with hearing loss
17	Peker et al. 2020. [59]	Case–control study	Experimental group (DHH): 34 DHH children and parents Control group (NH): 68 hearing children and parents	Did not mention	DHH: 19 males; 15 females NH: 36 males; 32 females	Child’s chronological age (months): DHH: 63.9 (11.7; 42–83) NH: 61.3 (10.1; 42–77)	All CI users	To compare children with CI and their parents with healthy children and their parents in terms of QoL and parental care burden
18	Piplani et al. 2022. [60]	Cross-sectional study	57 parents of children with CI	26 hearing mothers 31 hearing fathers	29 males 27 females	Age of deafness diagnosis (months): 18 (0.8; 4–did not mention); Child’s chronological age (years): 8.7 (4.2; did not mention); Child’s age of CI (years): 3.3 (1.6; 1.6–did not mention)	All CI users	To investigate the relationship between parental stress and attitude of parents toward the outcomes of cochlear implantation in a scenario in India
19	Saki et al. 2017. [61]	Cohort study	40 mothers of children below 7 years old who underwent CI surgery	40 hearing mothers	24 males 16 females	Child’s chronological age (months): Male: 36.8 (16.1; 13–65) Female: 34.3 (16.7; 12–76)	All CI users	To investigate the impact of hearing improvement (through the CI) on the psychological variables of happiness and self-esteem of mothers of DHH children
20	Sarant & Garrard. 2014. [62]	Cross-sectional study	70 parents of children with CI	68 hearing mothers 2 hearing fathers	36 males 34 females	Age of deafness diagnosis (months): 7.5 (7.5) Child’s chronological age (years): 6.6 (1.4; 5–8) [5 years old: *n* = 55; 8 years old: *n* = 25]) Child’s age of CI1 (months): 18.6 (9.6; did not mention–42) Child’s age of CI2 (months): did not mention (did not mention; did not mention–72)	16 unilateral CIs 54 bilateral CIs	To examine the stress experience of parents of children with CIs by comparing their stress levels to those of parents of children without disabilities, identifying primary stressors, examining the relationship between parents’ stress levels and child’s language, and comparing stress levels in parents of children with bilateral and unilateral CIs
21	Sipal & Sayin. 2013. [63]	Cross-sectional study	103 mothers of children with deafness	103 hearing mothers	50 males 53 females	Child’s chronological age (months): 54.6 (36–72) 36–48 months: 24 49–60 months: 39 61–72 months: 40	Did not mention	To assess the impact of having a child with DHH on maternal depression and examine how social support can facilitate coping with the depression caused by deafness as well as the parenting behaviors of those mothers
22	Talebi et al. 2018. [64]	Case–control study	50 parents (fathers and mothers) whose child had CI for 6 months 50 parents (fathers and mothers) of hearing children (NH)	CI: 25 hearing fathers and 25 hearing mothers NH: 24 hearing fathers and 26 hearing mothers	CI: 31 males and 19 females NH: 20 males and 30 females	Child’s chronological age (months): age range for children of both groups was the same (1–3 years old) age of CI: before 3 years	All CI users	To measure anxiety levels experienced by parents of children with CI and compare the anxiety levels experienced by parents of children with CI with those experienced by parents of children with NH

HA, hearing aid; BAHAs, bone-anchored hearing aids; CI, cochlear implants; DHH, deaf and hard of hearing; NH, normal hearing; ABI, auditory brainstem implant

^#^ This article included school-aged children (6–12 years old) who are not the participants of interest in the present study; therefore, the relevant information will not be included in this table.

^$^ These articles included children with other disabilities; however, the present study retains these articles as the study participants with hearing loss met the inclusion criteria, and these articles contain several validated instruments used for evaluating parental emotional status and ecological support systems.

## Results

### Selection of sources of evidence

Of the 3,060 articles retrieved from the databases (Medline OVID [685] and EMBSCO [1,631], Scopus [59], and WOS [685]), 2,680 were excluded owing to duplication of articles and articles’ subjects, abstracts, and/or title headings. Next, after reviewing 139 full-text articles, 117 articles were excluded for the following reasons: the age of the DHH children, either chronological or age at implantation, was not within 0–6 years, regardless of whether the hearing device was either bilateral hearing aids, bilateral CI, unilateral CI or bimodal (*n* = 51; 43.59%); did not contain validated instrument (*n* = 23; 19.66%); not related to the present study’s aims (e.g., outcomes were focused on children rather than parents, and outcomes were not related to PES or ESS; *n* = 30; 25.64%); the types of research designs did not conform to the study’s inclusion criteria (mentioned in the methodology; *n* = 7; 5.98%); the articles were not written in English (*n* = 4; 3.42%); did not provide child’s age-related information (*n* = 1; 0.85%); and the full-text cannot be accessed (*n* = 1; 0.85%). Overall, 22 articles met the inclusion criteria (Fig 1).

### Characteristics of the sources of evidence

The included articles were published from 2007 to 2022, and approximately half (10 articles) of them were published within five years (since 2019). In addition, most studies had cross-sectional designs (*n* = 14; 63.63%), whereas case-control study designs (*n* = 5; 22.73%) and cohort study designs (*n* = 3; 13.63%) comprised the minority. Of the included studies, four (18.18%) included parents of hearing children as the control group [45, 49, 59, 64]. Furthermore, one study that administered a qualitative instrument was included in the present study because it used a validated instrument and simultaneously interpreted the findings from both qualitative and quantitative approaches, which met the present study’s inclusion criteria [56]. Moreover, nine (40.9%) of the included studies focused on parents of children with at least a CI only, whereas only one study focused on parents of children who are CI candidates only. Moreover, six (27.3%) and two (9.1%) of the included studies comprised parents of children with different types of hearing devices and HA users only, respectively.

Of the parents of DHH children in the included studies, mothers represented approximately 54.4% (*n* = 947), whereas fathers represented only 25.4% (*n* = 443). Some studies referred to the responders as parents (*n* = 347; 19.9%) rather than specifying them as either fathers or mothers; therefore, the number did not count into either mothers or fathers [47, 49, 55, 59]. Moreover, among the study population, a portion of the children who experienced hearing loss were born to mothers (*n* = 3), fathers (*n* = 14), or parents (*n* = 5) with a history of hearing loss [49–51, 53]. Notably, a small percentage of grandparents participated in the study (*n* = 3; 0.2%), refer to Table 1 for details.

### Results of individual sources of evidence

Of the included studies, approximately 22 and 10 evaluated PES and ESS among parents of DHH children, respectively. Among them, different questionnaires were identified and targeted various related concepts (Table 2), including PES (number of instruments = 23) and ESS (number of instruments = 12). Except for the Clarke Modification of the Holroyd Questionnaire on Resources and Stress (Clarke QRS; 0.68), the Parental Involvement on Child Care Index (range 0.67–0.68), and the social relationships domain (0.66) of the World Health Organisation Quality of Life Questionnaire–Short Form (WHOQOL-BREF), the majority of the instruments demonstrated acceptable (> 0.70) to good (> 0.80) internal consistency reliability (Table 2). Moreover, only a few condition-specific instruments were developed and used for evaluating either PES or ESS among parents of children with CI, that is, the Parental Attitudes of Various Aspects of Cochlear Implantation (PAVACI) questionnaire [64] and Parenting Stress-CI modules-early childhood (EC) version [47] for PES, and Children with Cochlear Implants: Parental Perspectives Questionnaire (CCIPP; [65–69]) for both PES and ESS.

**Table 2:** Outcomes and instruments used related to parental emotional status and ecological support systems among the included studies.

No.	Study	Parental Emotional Status	Parental Support Systems	Outcomes
1	Aiello & Ferrari. 2015. [44]	Parental Stress Index-Short Form (PSI-SF [71]; 36 items; 5-point scale); ICR: 0.93	-	No differences in PSI-SF were observed between groups (between subjects) and the first and second assessments (within subjects). However, a trend that the experimental group had lower PSI-SF scores than the control group in all four domains for both assessments was noted. Only the age of mothers was negatively significantly correlated with parent–child dysfunctional interaction. No significant correlation was observed between socioeconomic classification and PSI-SF.
2	Aras et al. 2014. [45]	Health-Related Quality of Life-Short Form (HRQOL-SF [72]; 36 items; 2/3/5/6-point scale among different items); ICR: 0.78–0.94	-	(1) Normal group: mothers scored significantly worse on role physical (RP) and social functioning (SF) than fathers (*p* < 0.05); (2) DHH group: mothers scored significantly worse (*p* < 0.05) in almost all SF-36 health dimensions except for physical functioning (PF) and general health perception (GH); HL and Normal: the mothers of DHH children scored significantly worse than the mothers in the control group in all health dimensions (*p* < 0.05) except for role emotional (RE), whereas fathers of DHH children scored significantly worse than the fathers in the control group in SF, pain (BP), and general health perception (GH).
3	Brand et al. 2018. [46]	Child acceptance ([73]; 30 items; 5-point scale); ICR: 0.89 and 0.86 for mothers and fathers, respectively	Parental Involvement Questionnaire ([74]; 23 items; 5-point scale); ICR: 0.87 and 0.89 for mothers and fathers, respectively Support System Questionnaire ([75]; 23 items; 6-point scale)—Family Support Scale (FSS); ICR: 0.93	Parents’ child acceptance positively influenced their involvement. A significant positive actor effect of social support on the involvement of mothers and fathers; a significant interaction effect for partner effect with gender was observed in mothers only.
4	Cejas et al. 2021. [47]	Family Stress Scale (16 items; 5-point scale); ICR: 0.76–0.86	-	The top five stressors included (1) finances, (2) educational concerns, (3) discipline, (4) safety, and (5) communication. FSS scores were significantly negatively correlated with parental involvement and parental self-efficacy.
5	Cejas et al. 2022. [48]	Parenting Stress-CI modules–Early childhood version [48] (EC; 15 items; 4-point scale); ICR: 0.88 PSI-SF ([76]; 36 items; 6-point scale); ICR: 0.93	-	A significant and strong correlation was observed between the Parenting Stress-CI module EC version and the generic PSI-4 Total Stress scale. No associations were noted between demographic variables, including age, gender, ethnicity, or number of children living in the home, for any of the Parenting Stress-CI measures.
6	Dirks et al. 2016. [49]	Nijmegen Parenting Stress Index (NPSI; Dutch version of PSI [77]; 123 items; 6-point scale); ICR: 0.92–0.95	Multidimensional Scale of Perceived Social Support (MSPSS [78];12 items; 6-point scale); ICR: 0.88	No significant differences were observed on the stress levels of parents with a child with MHL or a hearing child; neither on the child-related nor on the parent-related stress factors in NPSI. Parents of children with MHL perceived less social support than parents of hearing children. Higher levels of parent-related stress were related to (1) lower language ability in the younger children, (2) more internalizing behavior problems, and (3) less perceived social support.
7	Dirks & Szarkowski, 2022. [50]	Fathers of Children with Developmental Challenges instrument (FCDC [44, 53, 79]; 20 items; 5-point scale); ICR: 0.89	Perceived Support by Family-Centered Early Intervention ([53]; 6 items; 5-point scale); ICR: 0.85	Although no significant differences in the mean score were noted between fathers and mothers in the FCDC, a significant number of fathers and mothers who experienced some struggles with having a child with DHH was observed in the item-level analysis. Both fathers and mothers perceived less support from their EI provider in one significant area—handling reactions from family, friends, and society regarding the child’s hearing loss. Child age and parental education level did not affect these results.
8	Gascon-Ramos et al. 2010. [51]	Trait Emotional Intelligence Questionnaire—Short Form (TEIQue-SF [80, 81]; 30 items; 7-point scale); ICR: 0.90	My Views on Services questionnaire (MVOS [82]; 22 items; 4-point scale on adequate amount and 5-point scale on satisfaction); ICR: 0.86–0.88	Audiologists and teachers of the child with deafness were the professionals who were the most frequently and permanently involved with parents. Health visitors and speech and language therapists were other prominent professionals. Importance on the Supporting Parents subscale at entry was moderately highly correlated with 6 and 12 months, with no significant differences between scores at entry and 6 months later or at entry and 12 months later. Mothers’ education levels did not differ on the Supporting a Deaf Child subscale; however, statistically significant differences were observed in the Supporting Parents subscale, that is, mothers with higher educational qualifications scored significantly lower than those with fewer qualifications. No significant relationship was noted between the significance on the Supporting a Deaf Child and Supporting Parents subscales and their TEIQue scores. Mother’s well-being showed a statistically significant association with satisfaction with the content of early intervention (Supporting Parents and a Deaf Child subscales).
9	Green. 2020. [52]	Depression Anxiety Stress Scale (DASS Short form [83]; 21 items; 4-point scale); ICR: 0.93	-	Depression and anxiety scores were lower and stress scores were higher than the norms in Australia. Higher levels of parental anxiety were associated with a reduction in the likelihood of early intervention service engagement.
10	Hintermair & Sarimski. 2019. [53]	FCDC ([79]; 20 items; 5-point scale); ICR: 0.89	Perceived Support by Early Intervention ([84]; 15 items; 5-point scale); ICR: 0.85 German version of the Quality of Marriage Index (QMI-D [85]; 6 items; first 5 items, 7-point scale; last item, 10-point scale); ICR: 0.94	Fathers seemed to cope relatively well with their situation as fathers of a child with DHH, and they showed readiness and strength to face the challenge of educating their child with deafness. Fathers who participated more frequently in early intervention appointments perceived receiving more support from early intervention services. Fathers who engaged more in daily childcare and were involved more in early intervention tended to report a satisfying partnership.
11	Jackson et al. 2010. [54]	Beach Center Family Quality of Life scale (FQoL [86]; 26 items; 5-point scale, with an open-ended question related to identifying family support the parents desired during early intervention); ICR: 0.88	FQoL [86]; ICR: 0.88	The mean score of the “emotional well-being” domain (3.65) was the lowest, whereas the mean scores of the other domains were >4.0 (satisfied). The “emotional well-being” domain demonstrated the largest impact of deafness, whereas the other domains reflected a small-to-moderate impact of deafness. Recommended support for parents was related to general emotional well-being, stress relief, counseling, and time to pursue individual interests. Desire for additional social network and parent support groups.
12	Kobosko et al. 2021. [24]	Self-Perception of Parental Role (S-PPR [87]; 22 items; 4-point scale); ICR: 0.72–0.80 Family Quality of Life Survey (FQOLS-2006 [88]; 54 items; 5-point scale); ICR: 0.89	FQOLS-2006 [88]; ICR: 0.89	No significant differences were observed in any of the FQOLS-2006 dimensions between families of DHH children with low- and medium-high development quotient (DQ). Families of children with deafness with low DQ have lower scores than the families of children with deafness with medium-high DQ in the investment domain (S-PPR).
13	Kushalnagar et al. 2007. [55]	Parenting Stress Index (PSI [89]; 101 items; 5-point scale); ICR: 0.80	-	The relationship between adaptive behavior and depression was significant; however, parental depression did not hold a significant contributory role in adaptive behavior after controlling for demographic variables that were correlated with adaptive behavior.
14	Majorano et al. 2020. [56]	PSI-SF ([76]; 36 items; 5-point scale); ICR: 0.93	-	Broad similarities or only slight decreases were noted between before and after CI activation. Moreover, the mothers showed that general stress levels declined after 6 months; however, the decline was not statistically significant. Parent–child dysfunctional interaction was significantly associated with the theme of the future, that is, having difficulty interacting with their children was correlated with a low number of themes focused on the child’s future.
15	Mavrogianni & Lampropoulou. 2020. [57]	Clarke Modification of the Holroyd Questionnaire on Resources and Stress (Clarke QRS [90]; 78 items; 3-point scale); ICR: 0.68	Family Support Scale (FSS [75]; 18 items; 5-point scale); ICR: 0.93 Kansas Marital Satisfaction Scale (KMSS [91]; 3 items; 7-point scale); ICR: 0.90 Parental Involvement on Child Care Index (PICCI [92]; 23 items, 21 of which were scored on a Likert-type scale, and 2 required fathers to report the percentage of time their partner and themselves had sole childcare responsibility); ICR: 0.67–0.68	Stress level: DHH < autism and intellectual disability; DHH > without disability Marital satisfaction: DHH > autism and intellectual disability; DHH = without disability Social support: DHH = autism and intellectual disability; DHH < without disability Involvement: no differences were noted among groups. Deaf: fathers’ involvement was significantly positively correlated with marital satisfaction. Marital satisfaction and stress had a statistically significant effect on involvement, and marital satisfaction of fathers of DHH children had a higher effect on their involvement than their stress as marital satisfaction had a higher standardized coefficient.
16	Munoz et al. 2015. [58]	Acceptance and Action Questionnaire (AAQ-II [93]; 7 items; 7-point scale); ICR: 0.84 Patient Health Questionnaire (PHQ-9 [94]; 9 items; 4-point scale); ICR: 0.89	-	Both mothers and fathers accepted their child’s hearing loss (*n* = 49; 89%); however, approximately one-fourth (*n* = 14; 26%) reported being concerned with the appearance of their child’s HA, and 16 (29%) were concerned about what others perceived. Overall responses indicated that most parents (96%) can healthily manage their internal emotions. Almost one-fourth of parents reported mild-to-severe symptoms of depression (*n* = 12; 22%; PHQ-9). Although mothers had a greater depression level than fathers, the difference did not reach a significant level. Almost half (*n* = 16; 40%) of the 40 parents who responded to the questionnaire indicated that depression was causing their managing ability to be somewhat or very difficult (mothers, *n* = 13; fathers, *n* = 3).
17	Peker et al. 2020. [59]	Zarit Burden Interview ([95]; 22 items; 5-point scale); ICR: 0.93 Children with Cochlear Implants: Parental Perspectives Questionnaire (CCIPP [67–70]; 58 items; 5-point scale); ICR: 0.89 World Health Organization Quality of Life Questionnaire–Short Form (WHOQOL-BREF [96]; 26 items; 5-point scale); ICR: 0.66–0.84	CCIPP ([67–70]; 58 items; 5-point scale); ICR: 0.89 WHOQOL-BREF ([95]; 26 items; 5-point scale); ICR: 0.66–0.84	Disease-specific QoL of the children with CI (CCIPP) was high in the general, support, social relationship, and clinical support subscales (≥70); however, their QoL related to the decision for implementation and positive effect of implant subscales was low (≤50). Other domains showed a moderate QoL among the parents. Parents of children with CI had a lower QoL in the “physical, mental, environmental, and social” domains and a higher care burden than those of children with NH (*p* < 0.05). WHOQOL-BREF domains were significantly negatively correlated with parental care burden.
18	Piplani et al. 2022. [60]	Parental stress scale ([97]; 18 items; 5-point scale); ICR: 0.80 Parental attitudes of various aspects of cochlear implantation (PAVACI) questionnaire ([65]; 70 items; 5-point scale); ICR: 0.89	-	No difference in stress levels was observed between fathers and mothers, and the parental stress among the parents was within the normal range. Parental stress was positively correlated with communication and social skills. Furthermore, CI duration was significantly correlated with communication and education.
19	Saki et al. 2017. [61]	Rosenberg Self-Esteem Scale ([98]; 10 items; 4-point scale); ICR: 0.77 Oxford Happiness Inventory ([99]; 29 items; 6-point scale); ICR: 0.91	-	Hearing CI surgery influenced the happiness and self-esteem scores of mothers of DHH children (before < after).
20	Sarant & Garrard. 2014. [62]	PSI ([89]; 151 items; 5-point scale); ICR: 0.80	-	Parents had a higher incidence of stress than the normative population. The level of support parents felt they received from sources outside the family was moderately correlated with the overall parents’ stress level, with parents who felt they received more support feeling less stressed. Moreover, a significant relationship was noted between how supported parents felt by their spouse and parents’ stress levels, with a high correlation between the amount of spousal support received and overall stress levels.
21	Sipal & Sayin. 2013. [63]	Beck Depression Inventory (BDI [100]; 20 items; 4-point scale); ICR: 0.90	MSPSS ([78]; 12 items; 7-point scale); ICR: 0.88	Of the mothers, 35 (33.9%) were below the clinical cutoff for depression, 41.7% showed increased depression scores (dysphoria), and 24.4% showed depression as indicated by a score of ≥10 (depression). Depression was negatively correlated with perceived family support. Support from family and friends predicted change in the depression scores of the mothers.
22	Talebi et al. 2018. [64]	Beck Anxiety Inventory (BAI [101]; 21 items; 4-point scale); ICR: 0.92	-	The parents of children with CI had significantly higher anxiety levels than those of children with NH. Of the parents, 8% (*n* = 4) reached the cutoff of moderate (2%) and severe (6%) levels of anxiety, which should be noticed.

ICR, internal consistency reliability; CI, cochlear implants; NH, normal hearing; HL, hearing loss; QoL, quality of life; MHL, moderate hearing loss; EI = early intervention; DHH, deaf and hard of hearing

#### Parental emotional status

Of the 23 instruments that evaluated PES, the Parenting Stress Index (PSI) developed by Abidin et al. [71, 76], in either the full-version (*n* = 3) or the short-form (*n* = 3), was the most used instrument (26.08%) for evaluating parental emotional-related indicators among parents of DHH children. The findings derived from the PSI-SF showed no statistically significant differences in parental stress levels experienced by parents of children with CI [56] or CI candidates [44] when their initial and subsequent evaluations were compared. Nevertheless, the findings from the PSI-full version yielded contrasting results, indicating that parents of children with CI exhibited higher stress levels than the normative population of the PSI [62], whereas parents of children with moderate hearing loss have similar stress levels comparable to the norms [49]. Moreover, social support, language, and internalizing behavior were significantly correlated with parental stress levels (PSI-full version; [49, 62]). When examining the domains, the theme of the child’s future [56] and the age of the mother [44] were significantly correlated with the “parent–child dysfunction interaction” domain in the PSI-SF, whereas no significant correlation was noted in the other domains.

Besides PSI, several instruments were used for assessing stress and anxiety levels among parents in the included studies. These instruments included the Beck Anxiety Inventory [64], Parental Stress Scale (PSS; [60]), Depression Anxiety Stress Scale (DASS; [52]), Health-Related Quality of Life–Short Form (HRQOL-SF; [45]), Parenting Stress–CI modules (EC; [48]), Trait Emotional Intelligence Questionnaire–Short Form (TEIQue-SF; [51]), Clarke QRS [57], Family Stress Scale [47], and Zarit Burden Interview [59]. Parents of DHH children showed significantly higher stress, anxiety, and care burden levels than those of hearing children [45, 52, 57, 59, 64]. Parents reported mild-to-severe depression and/or anxiety levels, indicating that they lacked sufficient emotional regulation skills [45, 58, 62–64]. Conversely, parental stress was significantly correlated with parental involvement among DHH children [47, 52, 57], indicating that parents with higher stress levels tend to have lesser involvement in their children’s intervention sessions. Additional comprehensive findings are presented in Table 2.

#### Ecological support systems

Of the 12 instruments that evaluated ESS, the Multidimensional Scale of Perceived Social Support (MSPSS; [49, 63]), Family Support Scale (FSS; [46, 57]), and the Perceived Support by Early Intervention scale [50, 53] were commonly used for assessing parental social support (ESS). Parents of DHH children [49, 57] perceived less social support than parents of children with typical hearing. Moreover, without adequate social support, parents exhibited less involvement in intervention sessions and experienced greater depression or stress levels [46, 49, 50, 53, 54, 57, 62, 63].

The findings obtained from the use of the “Beach Center Family Quality of Life Scale” (FQoL) suggested that parents expressed overall pleasure regarding all categories encompassed within the FQoL, except for the emotional well-being domain [54]. Based on the parents’ written comments, they evidently expressed a need for increased assistance in several aspects of family life and the overall well-being of the family. Furthermore, the families indicated a perceived requirement for assistance with respect to overall emotional well-being, stress relief, provision of counseling services, and time allocation for pursuing personal interests. Moreover, parents reported that the FQoL dimensions reflect the small-to-moderate impact of deafness, except for emotional well-being, which has the greatest impact on family life because of deafness [54]. Kobosko et al. [24] compared families of children with low development quotient to those with medium-to-high development quotient and reported no statistically significant differences in the quality of life of families with children who have bilateral severe or profound sensorineural hearing loss.

Furthermore, marital relationship was identified as a significant factor impacting parental involvement in childcare and early intervention sessions [53, 57]. Parents who were more engaged in daily childcare and early intervention sessions reported higher satisfaction levels in their partnerships, particularly among fathers of DHH children [53]. Notwithstanding, acknowledging that although the CCIPP scale was originally designed as a condition-specific measurement of a child with CI’s quality of life outcomes, certain domains within the scale pertain to the emotional well-being of the parents and support that the parents received, such as decision for implantation and process of implantation [59]. Consequently, these domains are included in the ensuing discussion. The analysis of the decision for implantation yielded a low score in terms of the parents’ quality of life, suggesting that they experienced significant stress during the decision-making process. However, the implantation process indicated a moderate quality of life, suggesting that the parents received sufficient assistance throughout the procedure [59]. Additional information is provided in Table 2.

## Discussion

### Summary of evidence

This scoping review ascertained suitable instruments for evaluating PES and ESS within the clinical context while also consolidating pivotal factors impacting PES and ESS in the context of early intervention for children with DHH children, especially for parents of children undergoing the CI journey. The current shortage of instruments adapted for parents of DHH children is evidenced by the limited number of studies included in this scoping review. Although numerous assessments are accessible to parents of children with CI, acknowledging that these instruments frequently incorporate challenging components that are mainly intended for post-CI only is needed.

### Why does evaluating parental emotional status and ecological support systems matter?

Seven (31.8%) of the articles included in the present analyses exclusively recruited individuals of a single gender, who were fathers only (*n* = 2; [53, 57]) and mothers only (*n* = 5; [24, 44, 56, 61, 63]). The representative participants’ distribution was unsurprising as mothers were frequently observed to be the child’s caregivers, and the fathers were frequently observed as breadwinners. Fathers showed low involvement with their children, considering that they were mostly invisible in the intervention sessions [29]. A study by Hintermair et al. [53] demonstrated that almost 76% of fathers were rarely involved in their child’s early intervention sessions with 15% of whom were not involved at all. Although studies have shown no significant correlation between fathers’ working hours and frequency of involvement in a child’s intervention [50, 53], most of the fathers mentioned that the main reason for being absent in their child’s intervention was the conflicting time schedule between the early intervention and their working hours [53]. However, fathers are no longer regarded as outsiders but as participants in child rearing, and their presence significantly impacts their child’s development [57, 73].

Hintermair et al. [53] reported that although fathers’ involvement may have been absent during the early intervention session, they remained a visible and integral part of the family-centered intervention framework. One of the key components of early intervention remains to be the engaged participation of family members [25]. Family member involvement plays a key role in family-centered interventions, whether it applies to selecting educational interventions for the child or making decisions regarding hearing interventions. This involvement includes choices such as total communication, sign language, or auditory–verbal interventions for educational interventions, and the selection of hearing aids or CIs for hearing interventions. The decision about CI surgery for children, particularly those who may benefit from it, frequently relies upon a thorough assessment conducted by professionals and adherence to certain implantation criteria. Nevertheless, the final decision about the pursuit of implantation often depends on the parents.

Further, several included studies indicated that PES significantly correlated with parental involvement in intervention sessions among parents of DHH children [28, 47, 52] and positively associated with their marital quality [29, 57]. In other words, parents with emotional stability, including low depression or stress levels, tend to engage more in their child’s intervention sessions, and providing emotional and behavioral support from the spouse would enhance their satisfaction with the marital relationship. A supportive spouse who not only participated in intervention sessions but also offered emotional support would establish an ideal home setting for children, thereby promoting their overall growth and progress. These study results may help researchers identify the indicators that may affect parents’ PES and ESS, which can be used as variables in future research to explore the causal relationship among parental involvement, marital relationship, and PES and ESS.

Emotionally stable parents receiving sufficient ESS during their child’s intervention journey can positively affect their child’s speech and hearing development [62]. However, this intervention journey is not a short-term effort but requires a long-term commitment from the parents. Committing to interventions that require constant involvement may be challenging for parents who do not have adequate ESS and stable PES [102, 103]. In addition, parents juggle multiple roles during the intervention journey, including being the child’s mother/father, teacher, case monitor, speech therapist, and driver, and must attend various interventional-related appointments that may cause exhaustion [103]. Negative emotions such as being overwhelmed, depressed, regretful, or helpless may emerge when a child fails to meet expectations or when the parents are blamed for missed appointments because of work schedules [30, 103]. These negative emotions can make it challenging for healthcare professionals to recommend appropriate interventions. Therefore, parents’ emotional preparedness and the adequacy of their ESS for their children’s intervention journey, particularly the CI journey, must be understood. The following section examines the relevant instruments for PES and ESS assessment in a clinical setting.

### Instruments for evaluating parental emotional status and ecological support systems evaluation among the included studies

The present review includes 22 studies, with 23 and 12 validated instruments used for evaluating PES and ESS, respectively, among parents of DHH children. Although several established validating instruments exist for evaluating PES and ESS in parents of DHH children, the available instruments mostly comprise generic measures that have been validated using evidence-based approaches, including the PSI (either short form [76] or full version [89]), WHOQOL-BREF [96], DASS [83], MSPSS [78], and others (Table 2; [26, 72, 73, 75, 80–83, 85–88, 90–95, 97–101]). These instruments can be employed for assessing either PES or ESS. However, generic methods possess the inherent capacity to exhibit reduced sensitivity or yield inconsistent outcomes when employed to assess certain populations or circumstances, particularly in PES [44, 49, 60]. As evidenced by the findings of this scoping review, no discernible disparity in parental stress levels was noted between parents of DHH children and parents of hearing children [49]. However, parents of children with CI experienced higher stress levels than parents of hearing population [59, 62, 64]. Nevertheless, upon closer examination of parental stress levels, the stress indices of these parents (both pre- and post-CI) remained within the fair range [44, 56, 60]. Notably, among the abovementioned studies, only two studies used condition-specific instruments for evaluating parental stress levels [59, 60]. Therefore, the observed disparities that appear substantial lack substantive significance.

Conversely, an interesting finding was observed regarding ESS. Parents of children with moderate hearing loss and CI experienced lower social support levels than parents of hearing children [49, 57]. Meanwhile, the findings indicated that parents who exhibit more emotional stability, characterized by lower depression, anxiety, and stress levels, or who are engaged in their child’s daily activities or intervention sessions, tend to receive greater assistance from their family, friends, or spouse [49, 53, 57, 62, 63]. However, acknowledging that some of the population and item design logic behind the instruments were not originally intended for children with disabilities, for instance, MSPSS [49, 63], which was devised by university undergraduate students [78], is significant. Consequently, critically examining and assessing their psychometric features before their use with the population with disabilities particularly those who are DHH are necessary.

### Condition-specific instruments for parents of children with CI

Owing to the irreversible nature of CI surgery, parents must have a positive emotional status and adequate ESS throughout their long-term CI journey (including pre- and post-CI surgery) to not only increase their involvement intention but also to facilitate their child’s spoken language development [30]. For example, although hearing aids did not benefit their children and the children kept removing them because of discomfort, parents still needed to ensure that their children consistently wore the hearing aids to meet the requirements for undergoing CI surgery. Dealing with their children’s uncooperativeness during this process was challenging and caused negative experiences for the parents. If the children have undergone CI surgery, parental involvement will affect the child’s speech and hearing development effectiveness [62, 104, 105]. Thus, parents should exert more effort to cope with mapping sessions and speech therapies, that is, they must attend all the appointments without affecting their daily routine. This can be a challenging task because they have to balance their work schedule, attend therapy sessions, and handle the financial cost of CI devices, which can be emotionally burdensome [106]. Therefore, for healthcare professionals who are working with families of children with CI, the condition-specific instrument design is important to acquire a better understanding of the families’ circumstances. Of note, although the present scoping review identified mostly generic measures among the instruments examined, few instruments were observed to be condition-specific measures, specifically designed for assessing parents of children with CI. Examples of such instruments include the CCIPP [67–70], PAVACI [65], and Parenting Stress-CI modules–EC [48].

The CCIPP scale contains several versions, each indicating slight variation in terms of domains and items [68–70]. The number of domains ranged from 8 to 11, whereas the number of items ranged from 48 to 74. In the study of Peker et al. [59] employed the instrument developed by Incesulu [70], and when compared with Archbold’s version [67, 68], it became apparent that Archbold’s version encompassed a larger quantity of items. In Archbold’s version, additional items were included in several domains to gain insight into the present condition of parents and children with CI, especially the parental-related domains (“decision making” and “process of implantation”), such as the parents’ potential hesitations or concerns regarding decision-making, the expected outcomes for their child following CI surgery, and the support they received from others. Nevertheless, of note, both versions primarily prioritize the assessment of the child’s quality of life rather than that of the parents, and the generalizability of these findings may be limited. For example, based on the findings of the “process of implantation” domain, whether parents expressed concerns regarding their child’s outcome or lacked sufficient support from others following CI surgery is difficult to ascertain. Besides, another limitation of using the CCIPP is that it was designed for parents whose children had CI for at least 3 years. This scale cannot be used to measure parental concerns and support systems among parents of children with CI candidates and newly implantees (within 1 year). Consequently, if healthcare professionals use the CCIPP to measure parental quality of life, its practical limitations and interpretive power may be questioned.

Similarly, to assess parental attitudes toward their children’s CI, the PAVACI instrument was developed [65]. This instrument comprises 70 items distributed across six different domains. A subtle distinction exists between the CCIPP and PAVACI scales in terms of the composition of the samples used during their respective development processes. The development of the PAVACI scale was specifically informed by data collected from a cohort of parents and children 1 year following CI surgery. However, although this scale demonstrates suitability in evaluating the outcomes of parents with newly implanted children (1 year of CI experience), its applicability may be limited when assessing parents whose children have not yet undergone CI surgery. Furthermore, half of the items, specifically 35 items, were related to parents’ support systems, encompassing several aspects, including services provided by the CI center, rehabilitation programs, and decision-making processes. Half of the items remained targeted at the child’s outcomes regarding the child’s communication, academics, social skills, and social abilities. Thus, the PAVICI scale’s content may not be appropriate for evaluating PES in relation to their child’s CI and their ESS during the CI journey. Therefore, in the context of clinical practice, individuals who lack proficiency in counseling, such as audiologists who frequently interact with families, encounter difficulties in understanding the parental emotions and the support they need.

Currently, the Parenting Stress-CI modules–EC (15 items) is considered the most suitable condition-specific instrument for evaluating the emotional status of parents who have children with CI [48]. The Parenting Stress-CI modules–EC demonstrates a greater connection and closeness to the familial circumstances of children with CI than the PSS or PSI scales. Specifically, the scale now includes items addressing concerns related to fear of losing the CI, managing the CI, or dealing with individuals staring at a child’s CI. These items emphasize the stress experienced by parents because of the appearance of the CI and the potential financial and managerial risks it encompasses. The published research demonstrated that the instrument shows favorable reliability and validity levels [48]. Also, the scale underwent a convergent validity test using the PSI-SF, and the findings were satisfactory, that is, the correlation between the two measures approached a significant level (*r* = .66, *p* < .001). Despite the inclusion of inquiries regarding potential CI-related risks, including financial costs and CI appearance, and the examination of the impact of family dynamics on parental stress levels, considering that the instrument still has its limitations is essential. Notably, the population recruitment during the instrument’s development phase may present certain limitations, as most participants were Caucasians. Therefore, the generalizability of the Parenting Stress-CI modules–EC to other ethnic groups remains uncertain. Furthermore, the scale employed in the Cejas et al. [48] study lacked different domains. Consequently, the interpretation of parental stress levels solely relied on the overall score, making it difficult to recognize specific stressors for families or parents. Specifically, without a detailed analysis, ascertaining the specific aspects (CI appearance, financial costs, family support, healthcare professionals’ support, and child’s expectation outcomes) that contribute to the experience of stress is challenging. Moreover, as the scale must be accompanied by social support-related instruments to understand the parent’s current emotional status and support systems, healthcare professionals should be aware of the limitations of the scale. Simultaneously, employing additional instruments pertaining to social support to comprehensively comprehend the prevailing emotional condition and support systems of parents of children with CI is imperative.

### Limitations

This scoping review skips over gray literature, such as unpublished works or theses, as well as peer-reviewed publications written in languages other than English, such as Mandarin and Malay. Considering the multi-lingual nature of Malaysia, the exclusion of Mandarin and Malay literature may result in a missed opportunity to include valuable information that can be used in academic and clinical contexts within the country. This exclusionary approach may result in the omission of studies relevant to the present study’s objectives. Furthermore, note that this scoping review did not evaluate the quality of the studies included in the analyses. However, all included articles were extracted from bibliographic databases and had undergone the rigorous process of peer review before publication. Lastly, based on the findings of previous studies, the present study identified various factors that could influence PES and ESS. Notably, key indicators such as ethnicity, economic status, area of living, and culture have not been thoroughly examined [107]. This suggests that the results of the included studies do not necessarily apply to Asian cultures or even multicultural Malaysians. Future studies should investigate these significant indicators to better understand how PES and ESS are affected across different populations.

## Conclusions

This scoping review summarized studies related to PES and ESS among parents of DHH children, particularly those children undergoing the CI journey, and identified suitable instruments for evaluating PES and their ESS. Previous studies have provided evidence of the positive effects of early intervention on the future developmental outcomes of children diagnosed with hearing loss. However, in the context of family-centered intervention, parents play a significant role in selecting intervention sessions for their children. When parents lack emotional preparedness and have inadequate social support, they may not possess the necessary knowledge to immediately seek assistance, which could delay enrolling their children in early intervention programs. Healthcare professionals must realize that parents of DHH children, particularly those who have undergone CI surgery, necessitate an adequate amount of time to understand and regulate their emotions, as well as reorganize their support systems. However, no condition-specific instruments for evaluating PES and ESS among parents of DHH children are available. Nevertheless, condition-specific instruments for evaluating PES and ESS among parents of children with CI are available; however, their applicability is limited. Therefore, if non-psychological experts (including audiologists and speech-language pathologists) in the CI team have suitable instruments to employ throughout their consultation session, they will not only provide services in monitoring children’s hearing status but also guide parents, as required. To develop a condition-specific instrument that could benefit healthcare professionals in identifying indicators of PES and ESS among parents of children with CI, further study is warranted. The instrument would enable healthcare professionals to offer appropriate assistance to the parents, potentially leading to a timelier intervention.

## Supporting information

S1 Checklist Preferred Reporting Items for Systematic reviews and Meta-Analyses extension for Scoping Reviews (PRISMA-ScR) checklist. (DOCX)
